# Supplementary material for: Hedgehogs and Squirrels as Hosts of Zoonotic Bartonella Species
Source: Pathogens. 2021 Jun 1;10(6):686. doi: 10.3390/pathogens10060686 (PMC8229113; doi:10.3390/pathogens10060686)

Figure S1: Comparison of the portion of particular tissue samples with the lowest Cp value as assessed by real-time PCR in *Bartonella*-positive animals (verified by conventional PCRs)

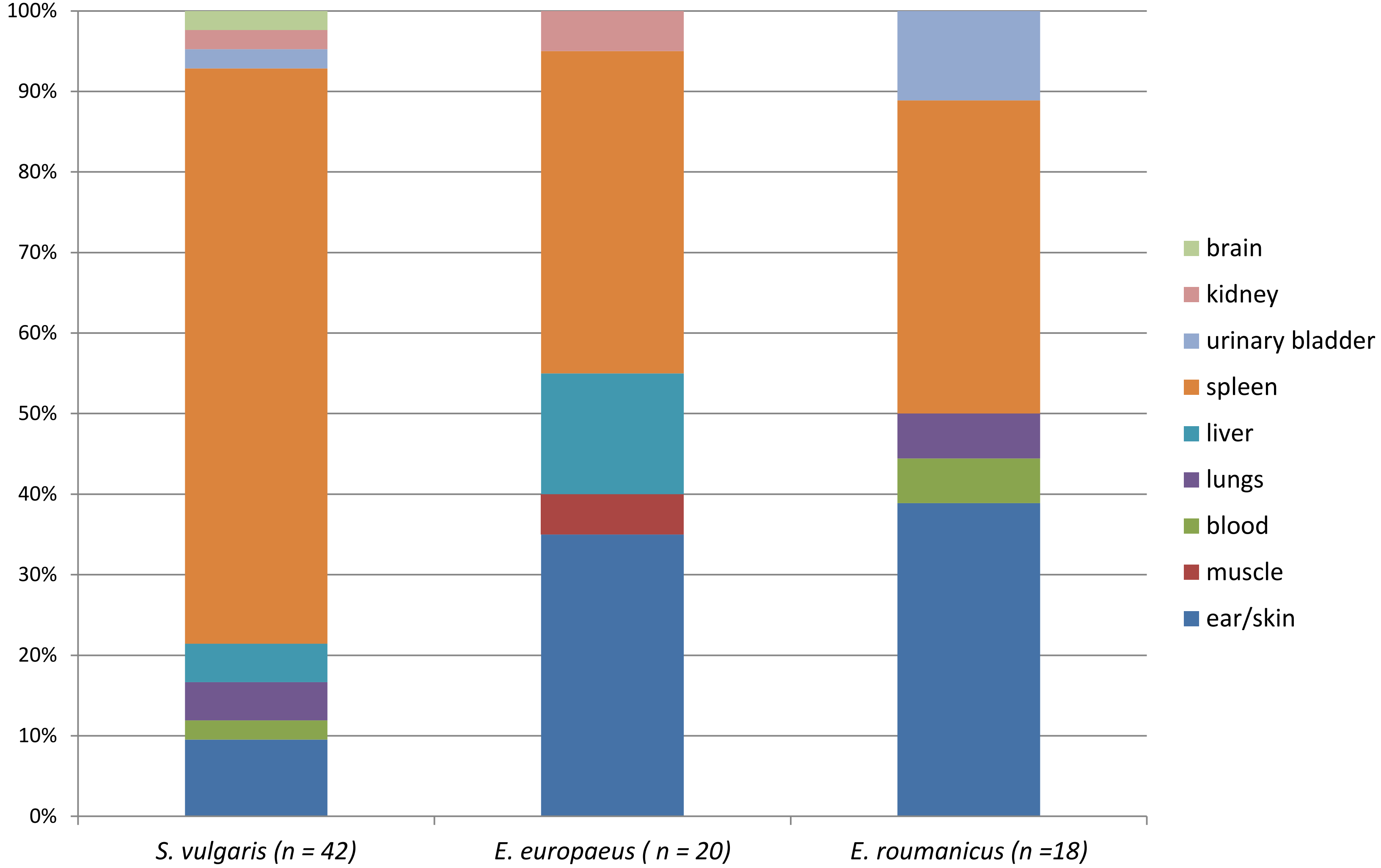

Supplement: Supplementary file 1 [file pathogens-10-00686-s001.zip › Supplementary files/Figure S1 Comparison of the portion of particular tissue samples with the lowest Cp value_NEW.pdf]
