# Supplementary material for: Hedgehogs and Squirrels as Hosts of Zoonotic Bartonella Species
Source: Pathogens. 2021 Jun 1;10(6):686. doi: 10.3390/pathogens10060686 (PMC8229113; doi:10.3390/pathogens10060686)
Supplement: Supplementary file 1 [file pathogens-10-00686-s001.zip › Supplementary files/Table S1 Overview of the occurence of zoonotic Bartonella spp. in humans_FINAL with references.pdf]

Table S1: Overview of the occurrence of zoonotic *Bartonella* spp. in humans

species	country	number of cases	described main symptoms	references
B. alsatica	France	3	endocarditis, lymphadenopathy	Raoult *et al*., 2006; Angelakis *et al*., 2008; Jeanclaude *et al*., 2009; Edouard *et al*., 2015
B. ancashensis	Peru	2	verruga peruana	Mullins *et al*., 2013; Mullins *et al*., 2015
B. clarridgeiae	USA, Germany, Brazil, Ireland	31	asymptomatic, endocarditis, lymphadenopathy, chest-wall abscess	Kordick *et al*., 1997; Margileth and Baehren, 1998; Sander *et al*., 2000; Vieria-Damiani *et al*., 2015; Logan *et al*., 2019
B. doshiae	France	1	fatigue, blurred vision, arthralgia	Vayssier-Taussat *et al*., 2016
B. elizabethae	USA, Thailand, Nepal, Mexico, Germany	8	endocarditis, neuroretinitis, febrile illness, bacillary angiomatosis, angioedema	Daly *et al*., 1993; O´Halloran *et al*., 1998; Kosoy *et al*., 2010; Myint *et al*., 2011; Lösch and Wank, 2014; Corral *et al*., 2019
B. grahamii	Netherlands, France, Finland	3	neuroretinitis, retinal artery occlusion, lymphadenopathy	Kerkhoff *et al*., 1999; Serratrice *et al*., 2003; Oksi *et al*., 2013
B. koehlerae	Israel, USA,	11	endocarditis, fatigue, insomnia, joint pain, headache, memory loss, muscle pain, rheumatologic and orthopedic symptoms	Avidor *et al*., 2004; Breitschwerdt *et al*., 2010a; Breitschwerdt *et al*., 2011; Mozayeni *et al*., 2018
B. kosoyi	Georgia	1	lymphadenopathy, fever	Kandelaki *et al*., 2016
B. mayotimonensis	USA	1	endocarditis	Lin *et al*., 2010
B. melophagi	USA	2	pericarditis, dry cough, fatigue, muscle pain	Maggi *et al*., 2009
B. rattimassiliensis	Thailand	1	febrile illness	Kosoy *et al*., 2010
B. rochalimae	Peru	2	fever, splenomegaly, Carrión disease symptoms	Eremeeva *et al*., 2007; Mujica *et al*., 2014
B. schoenbuchensis	France	1	fatigue, muscle pain, fever	Vayssier-Taussat *et al*., 2016
B. tamiae	Thailand, Nepal	5	febrile illness, fatigue, myalgia, headache, maculopapular rash, pterygium	Kosoy *et al*., 2008; Kosoy *et al*., 2010; Myint *et al*., 2011
B. tribocorum	Thailand, France	2	febrile illness, fatigue, muscle pain, headache	Kosoy *et al*., 2010; Vayssier-Taussat *et al*., 2016
***B. vinsonii* (subsp. not identified)**	France	1	lymphadenopathy	Rolain *et al*., 2009
B. vinsonii* subsp. *arupensis	France, Thailand, Russia, Nepal	14	endocarditis, febrile illness	Fenollar *et al*., 2005; Kosoy *et al*., 2010; Kirillov *et al*., 2007; Myint *et al*., 2011; Bai *et al*., 2012
B. vinsonii* subsp. *berkhoffii	France, USA, United Kingdom	17	endocarditis, fatigue, headache, muscle pain, blurred vision, neurological dysfunction	Roux *et al*., 2000; Breitschwerdt *et al*., 2007; Breitschwerdt *et al*., 2008; Breitschwerdt *et al*., 2010a; Olarte *et al*., 2012; Edouard *et al*., 2015; Breitschwerdt *et al*., 2010b
B. vinsonii* subsp. *vinsonii	Thailand, USA	2	febrile illness	Kosoy *et al*., 2010; Breitschwerdt and Maggi, 2019
B. washoensis	USA, Germany	3	endocarditis, fever, myocarditis, meningitis	Kosoy *et al*., 2003; Probert *et al*., 2009; von Loewenich *et al*., 2019

References:

Angelakis, E.; Lepidi, H.; Canel, A.; Rispal, P.; Perraudeau, F.; Barre, I.; Rolain, J.-M.; Raoult, D. Human Case of *Bartonella alsatica* Lymphadenitis. *Emerg. Infect. Dis.* **2008**, *14*, 1951–1953, doi:10.3201/eid1412.080757.

Avidor, B.; Graidy, M.; Efrat, G.; Leibowitz, C.; Shapira, G.; Schattner, A.; Zimhony, O.; Giladi, M. *Bartonella koehlerae*, a New Cat-Associated Agent of Culture-Negative Human Endocarditis. *J. Clin. Microbiol.* **2004**, *42*, 3462–3468, doi:10.1128/jcm.42.8.3462-3468.2004.

Bai, Y.; Kosoy, M.Y.; Diaz, M.H.; Winchell, J.; Baggett, H.; Maloney, S.A.; Boonmar, S.; Bhengsri, S.; Sawatwong, P.; Peruski, L.F. *Bartonella vinsonii* subsp. *arupensisin* Humans, Thailand. *Emerg. Infect. Dis.* **2012**, *18*, 989–991, doi:10.3201/eid1806.111750.

Breitschwerdt, E.B.; Maggi, R.; Duncan, A.W.; Nicholson, W.L.; Hegarty, B.C.; Woods, C.W. *Bartonella* Species in Blood of Immunocompetent Persons with Animal and Arthropod Contact. *Emerg. Infect. Dis.* **2007**, *13*, 938–941, doi:10.3201/eid1306.061337.

Breitschwerdt, E.B.; Maggi, R.; Nicholson, W.L.; Cherry, N.A.; Woods, C.W. *Bartonella* sp. Bacteremia in Patients with Neurological and Neurocognitive Dysfunction. *J. Clin. Microbiol.* **2008**, *46*, 2856–2861, doi:10.1128/jcm.00832-08.

Breitschwerdt, E.B.; Maggi, R.G.; Mozayeni, B.R.; Hegarty, B.C.; Bradley, J.M.; Mascarelli, P.E. PCR amplification of *Bartonella koehlerae* from human blood and enrichment blood cultures. *Parasites Vectors* **2010a**, *3*, 76, doi:10.1186/1756-3305-3-76.

Breitschwerdt, E.B.; Maggi, R.G.; Lantos, P.M.; Woods, C.W.; Hegarty, B.C.; Bradley, J.M. *Bartonella vinsonii* subsp. *berkhoffii* and *Bartonella henselae* bacteremia in a father and daughter with neurological disease. *Parasites Vectors* **2010b**, *3*, 29–9, doi:10.1186/1756-3305-3-29.

Breitschwerdt, E.B.; Mascarelli, P.E.; Schweickert, L.A.; Maggi, R.; Hegarty, B.C.; Bradley, J.M.; Woods, C.W. Hallucinations, sensory neuropathy, and peripheral visual deficits in a young woman infected with *Bartonella koehlerae*. *J. Clin. Microbiol.* **2011**, *49*, 3415–3417, doi:10.1128/jcm.00833-11.

Breitschwerdt, E.B.; Maggi, R.G. *Bartonella quintana* and *Bartonella vinsonii* subsp. *vinsonii* bloodstream co-infection in a girl from North Carolina, USA. *Med. Microbiol. Immunol.* **2019**, *208*, 101–107, doi:10.1007/s00430-018-0563-0.

Corral, J.; Robles, A.M.; Caire, S.T.; Hernández-Castro, R.; Moreno-Coutiño, G. First Report of Bacillary Angiomatosis by *Bartonella elizabethae* in an HIV-Positive Patient. *Am. J. Dermatopathol.* **2019**, *41*, 750–753, doi:10.1097/dad.0000000000001439.

Daly, J.S.; Worthington, M.G.; Brenner, D.J.; Moss, C.W.; Hollis, D.G.; Weyant, R.S.; Steigerwalt, A.G.; Weaver, R.E.; Daneshvar, M.I.; O'Connor, S.P. *Rochalimaea elizabethae* sp. nov. isolated from a patient with endocarditis. *J. Clin. Microbiol.* **1993**, *31*, 872–881, doi:10.1128/jcm.31.4.872-881.1993.

Drancourt, M.; Berger, P.; Terrada, C.; Bodaghi, B.; Conrath, J.; Raoult, D.; LeHoang, P. High Prevalence of Fastidious Bacteria in 1520 Cases of Uveitis of Unknown Etiology. *Medicine* **2008**, *87*, 167–176, doi:10.1097/md.0b013e31817b0747.

Eremeeva, M.E.; Gerns, H.L.; Lydy, S.L.; Goo, J.S.; Ryan, E.T.; Mathew, S.S.; Ferraro, M.J.; Holden, J.M.; Nicholson, W.L.; Dasch, G.A.; et al. Bacteremia, Fever, and Splenomegaly Caused by a Newly Recognized *Bartonella* Species. *N. Engl. J. Med.* **2007**, *356*, 2381–2387, doi:10.1056/nejmoa065987.

Fenollar, F.; Sire, S.; Raoult, D. *Bartonella vinsonii* subsp. *arupensis* as an Agent of Blood Culture-Negative Endocarditis in a Human. *J. Clin. Microbiol.* **2005**, *43*, 945–947, doi:10.1128/jcm.43.2.945-947.2005.

Jeanclaude, D.; Godmer, P.; Leveiller, D.; Pouedras, P.; Fournier, P.-E.; Raoult, D.; Rolain, J.-M. *Bartonella alsatica* endocarditis in a French patient in close contact with rabbits. *Clin. Microbiol. Infect.* **2009**, *15*, 110–111, doi:10.1111/j.1469-0691.2008.02187.x.

Kandelaki, G.; Malania, L.; Bai, Y.; Chakvetadze, N.; Katsitadze, G.; Imnadze, P.; Nelson, C.; Harrus, S.; Kosoy, M. Human Lymphadenopathy Caused by *Ratborne Bartonella*, Tbilisi, Georgia. *Emerg. Infect. Dis.* **2016**, *22*, 544–546, doi:10.3201/eid2203.151823.

Kerkhoff, F.T.; Bergmans, A.M.C.; Van Der Zee, A.; Rothova, A. Demonstration of *Bartonella grahamii* DNA in Ocular Fluids of a Patient with Neuroretinitis. *J. Clin. Microbiol.* **1999**, *37*, 4034–4038, doi:10.1128/jcm.37.12.4034-4038.1999.

Kirillov, M.I.; Markov, A.P.; Lopyrev, I.V.; Pankratova, V.N.; Levitskiĭ, S.A.; Bashkirov, V.N.; Smirnov, G.B.; Kruglov, A.N.; Osadchaia, V.A.; Frolova, G.P.; et al. Molecular genetic methods of typing of the *Bartonellae*. *Mol. Genet. Microbiol. Virol.* **2007**, *1*, 8–15.

Kordick, D.L.; Hilyard, E.J.; Hadfield, T.L.; Wilson, K.H.; Steigerwalt, A.G.; Brenner, D.J.; Breitschwerdt, E.B. *Bartonella clarridgeiae*, a newly recognized zoonotic pathogen causing inoculation papules, fever, and lymphadenopathy (cat scratch disease). *J. Clin. Microbiol.* **1997**, *35*, 1813–1818, doi:10.1128/jcm.35.7.1813-1818.1997.

Kosoy, M.; Murray, M.; Gilmore, J.R.D.; Bai, Y.; Gage, K.L. *Bartonella* Strains from Ground Squirrels Are Identical to *Bartonella washoensis* Isolated from a Human Patient. *J. Clin. Microbiol.* **2003**, *41*, 645–650, doi:10.1128/jcm.41.2.645-650.2003.

Kosoy, M.; Morway, C.; Sheff, K.W.; Bai, Y.; Colborn, J.; Chalcraft, L.; Dowell, S.F.; Peruski, L.F.; Maloney, S.A.; Baggett, H.; et al. *Bartonella tamiae* sp. nov., a Newly Recognized Pathogen Isolated from Three Human Patients from Thailand. *J. Clin. Microbiol.* **2007**, *46*, 772–775, doi:10.1128/jcm.02120-07.

Kosoy, M.; Peruski, L.F.; Maloney, S.A.; Boonmar, S.; Sitdhirasdr, A.; Lerdthusnee, K.; Baggett, H.; Morway, C.; Bai, Y.; Sheff, K.; et al. Identification of *Bartonella* Infections in Febrile Human Patients from Thailand and Their Potential Animal Reservoirs. *Am. J. Trop. Med. Hyg.* **2010**, *82*, 1140–1145, doi:10.4269/ajtmh.2010.09-0778.

Lin, E.Y.; Tsigrelis, C.; Baddour, L.M.; Lepidi, H.; Rolain, J.-M.; Patel, R.; Raoult, D. Candidatus *Bartonella mayotimonensis* and Endocarditis. *Emerg. Infect. Dis.* **2010**, *16*, 500–503, doi:10.3201/eid1603.081673.

Logan, J.M.J.; Hall, J.L.; Chalker, V.J.; O'Connell, B.; Birtles, R.J. *Bartonella clarridgeiae* infection in a patient with aortic root abscess and endocarditis. *Access Microbiol.* **2019**, *1*, e000064, doi:10.1099/acmi.0.000064.

Lösch, B.; Wank, R. Life-threatening angioedema of the tongue: The detection of the RNA of *B. henselae* in the saliva of a male patient and his dog as well as of the DNA of three *Bartonella* species in the blood of the patient. *BMJ Case Rep.* **2014**, *2014*, 2013203107, doi:10.1136/bcr-2013-203107.

Maggi, R.; Kosoy, M.; Mintzer, M.; Breitschwerdt, E.B. Isolation of *Candidatus Bartonella melophagi* from Human Blood1. *Emerg. Infect. Dis.* **2009**, *15*, 66–68, doi:10.3201/eid1501.081080.

Margileth, A.M.; Baehren, D.F. Chest-Wall Abscess Due to Cat-Scratch Disease (CSD) in an Adult with Antibodies to *Bartonella clarridgeiae*: Case Report and Review of the Thoracopulmonary Manifestations of CSD. *Clin. Infect. Dis.* **1998**, *27*, 353–357, doi:10.1086/514671.

Mozayeni, B.R.; Maggi, R.; Bradley, J.M.; Breitschwerdt, E.B. Rheumatological presentation of *Bartonella koehlerae* and *Bartonella henselae* bacteremias. *Medicine* **2018**, *97*, e0465, doi:10.1097/md.0000000000010465.

Mujica, G.M.; León, D.F.; Espinoza-Culupú, A. Identificación de *Bartonella rochalimae* en un Brote de Enfermedad de Carrión Mediante Caracterización Molecular del Gen 16S rRNA. In Proceedings of the VIII Congreso Internacional del Instituto Nacional de Salud, Lima, Peru,6 November 2014.

Mullins, K.E.; Hang, J.; Jiang, J.; Leguia, M.; Kasper, M.R.; Maguiña, C.; Jarman, R.G.; Blazes, D.L.; Richards, A.L. Molecular Typing of "Candidatus *Bartonella ancashi*," a New Human Pathogen Causing Verruga Peruana. *J. Clin. Microbiol.* **2013**, *51*, 3865–3868, doi:10.1128/jcm.01226-13.

Mullins, K.E.; Hang, J.; Jiang, J.; Leguia, M.; Kasper, M.R.; Ventosilla, P.; Maguiña, C.; Jarman, R.G.; Blazes, D.; Richards, A.L. Description of *Bartonella ancashensis* sp. nov., isolated from the blood of two patients with verruga peruana. *Int. J. Syst. Evol. Microbiol.* **2015**, *65*, 3339–3343, doi:10.1099/ijsem.0.000416.

Myint, K.S.A.; Gibbons, R.V.; Iverson, J.; Shrestha, S.K.; Pavlin, J.A.; Mongkolsirichaikul, D.; Kosoy, M.Y. Serological response to *Bartonella* species in febrile patients from Nepal. *Trans. R. Soc. Trop. Med. Hyg.* **2011**, *105*, 740–742, doi:10.1016/j.trstmh.2011.08.002.

O'Halloran, H.S.; Draud, F.; Be, M.K.M.; Rivard, A.K.; Pearson, P.A. Leber's neuroretinitis in a patient with serologic evidence of *Bartonella elizabethae*. *Retina* **1998**, *18*, 276–278, doi:10.1097/00006982-199803000-00015.

Oksi, J.; Rantala, S.; Kilpinen, S.; Silvennoinen, R.; Vornanen, M.; Veikkolainen, V.; Eerola, E.; Pulliainen, A.T. Cat Scratch Disease Caused by *Bartonella grahamii* in an Immunocompromised Patient. *J. Clin. Microbiol.* **2013**, *51*, 2781–2784, doi:10.1128/jcm.00910-13.

Olarte, L.; Ampofo, K.; Thorell, E.A.; Sanderson, S.; Doby, E.; Pavia, A.T.; Rosado, H.; Raoult, D.; Socolovschi, C.; Hersh, A.L. *Bartonella vinsonii* Endocarditis in an Adolescent With Congenital Heart Disease. *Pediatr. Infect. Dis. J.* **2012**, *31*, 531–534, doi:10.1097/inf.0b013e31824ba95a.

Pierre-Edouard, F.; Nabet, C.; Lepidi, H.; Fournier, P.-E.; Raoult, D. Bartonella, a Common Cause of Endocarditis: A Report on 106 Cases and Review. *J. Clin. Microbiol.* **2015**, *53*, 824–829, doi:10.1128/jcm.02827-14.

Probert, W.; Louie, J.K.; Tucker, J.R.; Longoria, R.; Hogue, R.; Moler, S.; Graves, M.; Palmer, H.J.; Cassady, J.; Fritz, C.L. Meningitis Due to a *Bartonella washoensis*-Like Human Pathogen. *J. Clin. Microbiol.* **2009**, *47*, 2332–2335, doi:10.1128/jcm.00511-09.

Puges, M.; Ménard, A.; Berard, X.; Geneviève, M.; Pinaquy, J.-B.; Edouard, S.; Pereyre, S.; Cazanave, C. An unexpected case of *Bartonella alsatica* prosthetic vascular graft infection. *Infect. Drug Resist.* **2019**, *12*, 2453–2456, doi:10.2147/idr.s206805.

Raoult, D.; Roblot, F.; Rolain, J.-M.; Besnier, J.-M.; Loulergue, J.; Bastides, F.; Choutet, P. First Isolation of *Bartonella alsatica* from a Valve of a Patient with Endocarditis. *J. Clin. Microbiol.* **2006**, *44*, 278–279, doi:10.1128/jcm.44.1.278-279.2006.

Rolain, J.; Boureau-Voultoury, A.; Raoult, D. Serological evidence of *Bartonella vinsonii* lymphadenopathies in a child bitten by a dog. *Clin. Microbiol. Infect.* **2009**, *15*, 122–123, doi:10.1111/j.1469-0691.2008.02197.x.

Roux, V.; Eykyn, S.J.; Wyllie, S.; Raoult, D. *Bartonella vinsonii* subsp. *berkhoffii* as an Agent of Afebrile Blood Culture-Negative Endocarditis in a Human. *J. Clin. Microbiol.* **2000**, *38*, 1698–1700, doi:10.1128/jcm.38.4.1698-1700.2000.

Sander, A.; Zagrosek, A.; Bredt, W.; Schiltz, E.; Piémont, Y.; Lanz, C.; Dehio, C. Characterization of *Bartonella clarridgeiae* Flagellin (*FlaA*) and Detection of Antiflagellin Antibodies in Patients with Lymphadenopathy. *J. Clin. Microbiol.* **2000**, *38*, 2943–2948, doi:10.1128/jcm.38.8.2943-2948.2000.

Serratrice, J.; Rolain, J.-M.; Granel, B.; Ene, N.; Conrath, J.; Avierinos, J.-F.; Disdier, P.; Raoult, D.; Weiller, P.J. Occlusion bilatérale des branches de l'artère centrale de la rétine révélant une infection à *Bartonella grahamii*. *La Revue Méd. Interne* **2003**, *24*, 629–630, doi:10.1016/s0248-8663(03)00224-8.

Terrada, C.; Bodaghi, B.; Conrath, J.; Raoult, D.; Drancourt, M. Uveitis: An emerging clinical form of *Bartonella* infection. *Clin. Microbiol. Infect.* **2009**, *15*, 132–133, doi:10.1111/j.1469-0691.2008.02202.x.

Vayssier-Taussat, M.; Moutailler, S.; Féménia, F.; Raymond, P.; Croce, O.; La Scola, B.; Fournier, P.-E.; Raoult, D. Identification of Novel Zoonotic Activity of *Bartonella* spp., France. *Emerg. Infect. Dis.* **2016**, *22*, 457–462, doi:10.3201/eid2203.150269.

Vieira-Damiani, G.; Diniz, P.P.V.D.P.; Pitassi, L.H.U.; Sowy, S.; Scorpio, D.G.; Lania, B.G.; Drummond, M.R.; Soares, T.C.B.; Barjas-Castro, M.D.L.; Breitschwerdt, E.; et al. *Bartonella clarridgeiae* Bacteremia Detected in an Asymptomatic Blood Donor. *J. Clin. Microbiol.* **2014**, *53*, 352–356, doi:10.1128/jcm.00934-14.

Von Loewenich, F.D.; Seckert, C.; Dauber, E.; Kik, M.J.L.; De Vries, A.; Sprong, H.; Buschmann, K.; Aardema, M.L.; Brandstetter, M. Prosthetic Valve Endocarditis with *Bartonella washoensis* in a Human European Patient and Its Detection in Red Squirrels (*Sciurus vulgaris*). *J. Clin. Microbiol.* **2019**, *58*, 01404–01419, doi:10.1128/jcm.01404-19.
