# Supplementary figures and images for: Hedgehogs and Squirrels as Hosts of Zoonotic Bartonella Species
Source: Pathogens. 2021 Jun 1;10(6):686. doi: 10.3390/pathogens10060686 (PMC8229113; doi:10.3390/pathogens10060686)

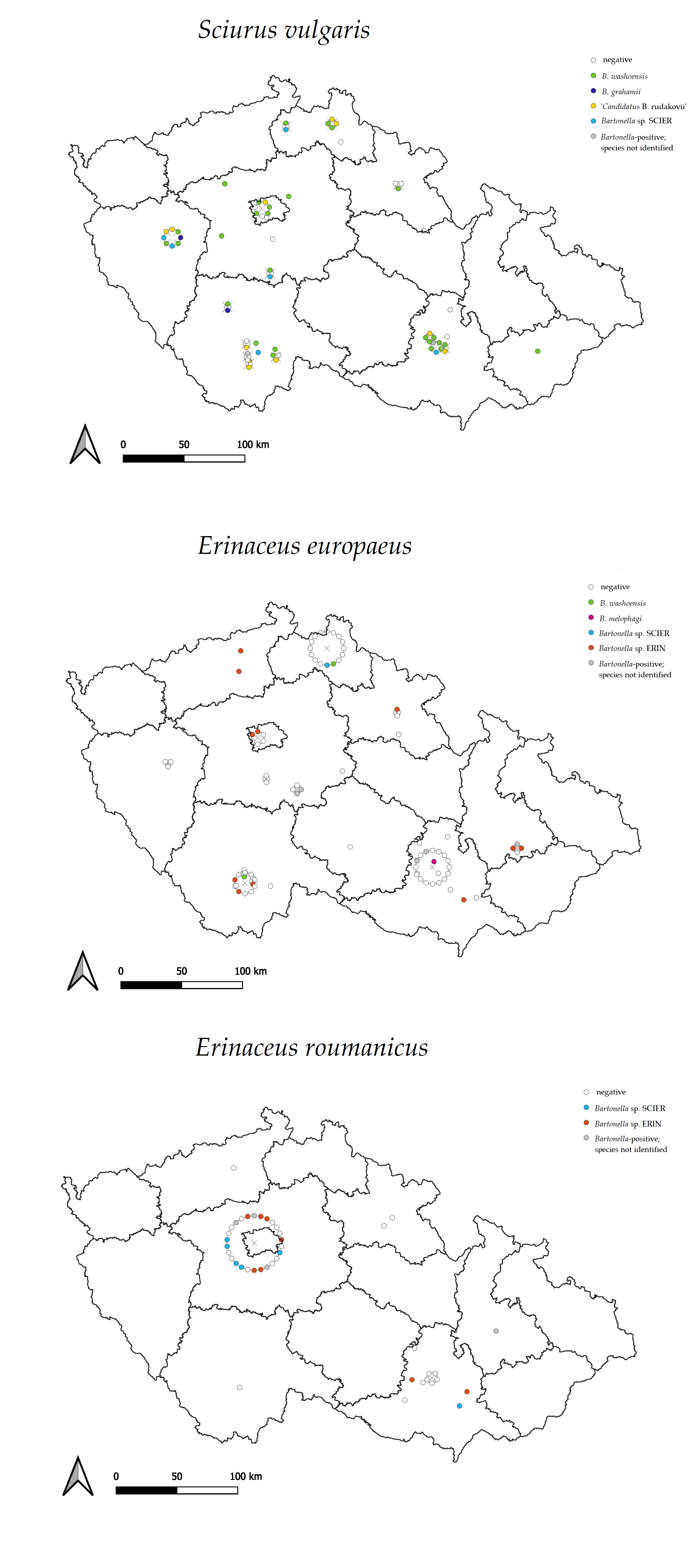

Supplement: Supplementary file 1 [file pathogens-10-00686-s001.zip › Supplementary files/Figure S2 maps_ACTUAL.png]

Figure S3A

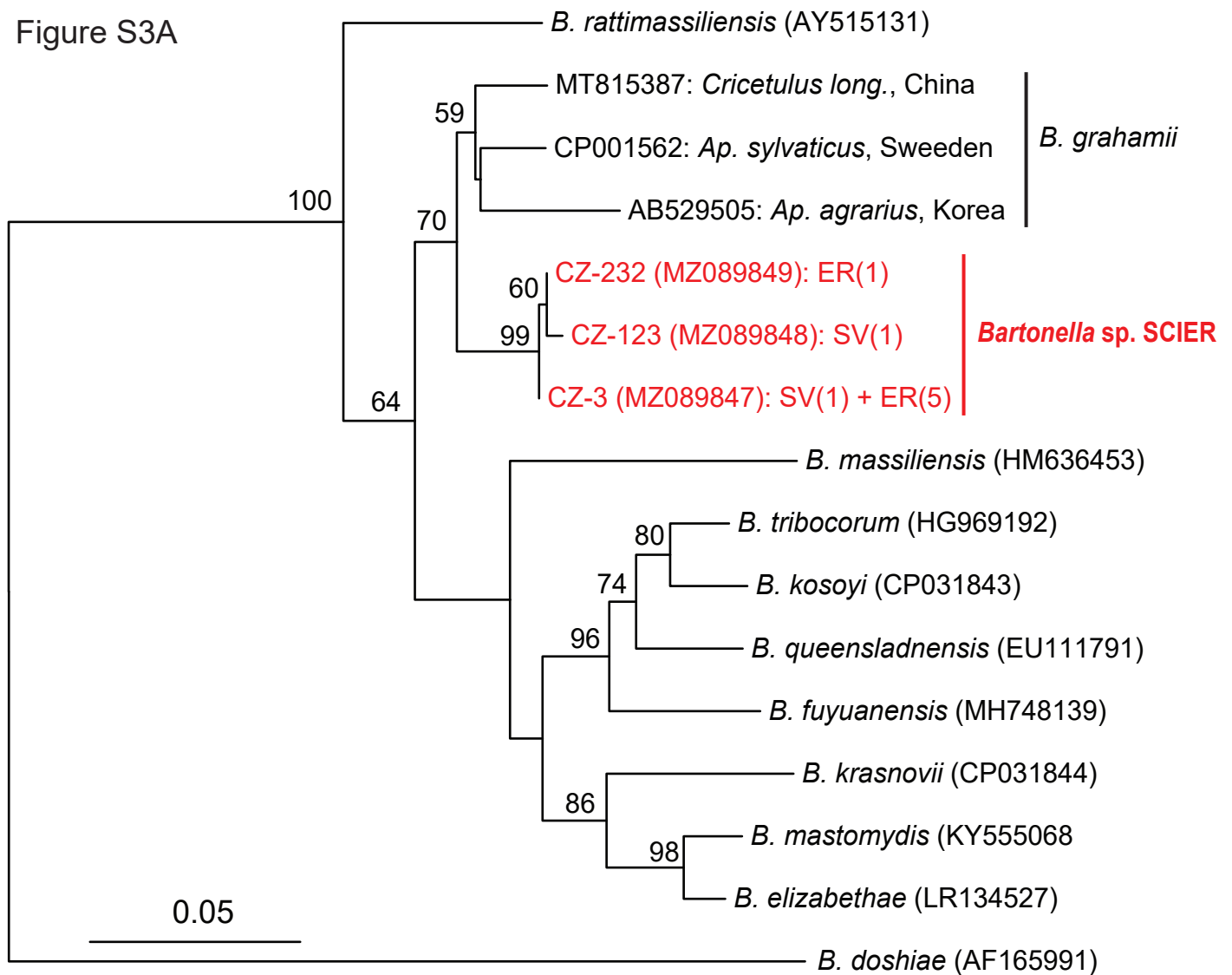

Figure S3B

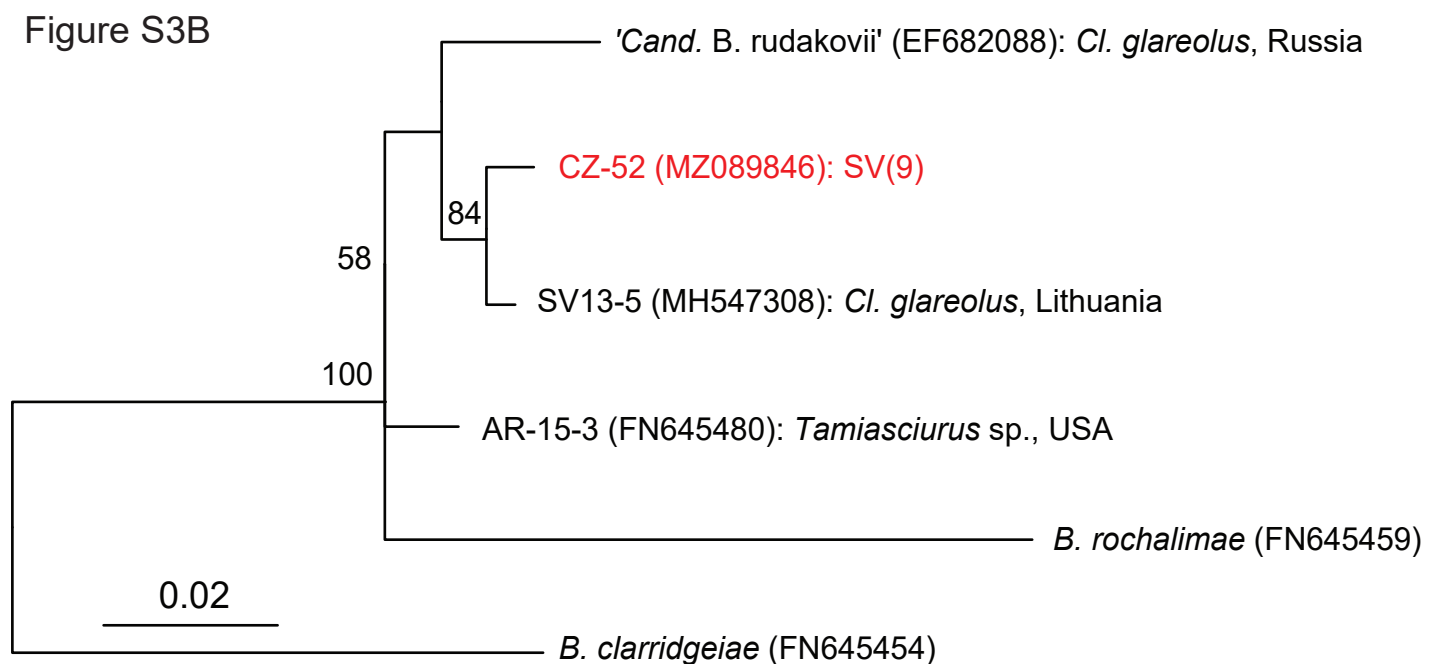

Supplement: Supplementary file 1 [file pathogens-10-00686-s001.zip › Supplementary files/Figure S3 trees rpoB with GB numbers.pdf]
